# Absence of the Non-Signalling Chemerin Receptor CCRL2 Exacerbates Acute Inflammatory Responses *In Vivo*

**DOI:** 10.3389/fimmu.2017.01621

**Published:** 2017-11-21

**Authors:** Daniel Regan-Komito, Sophia Valaris, Theodore S. Kapellos, Carlota Recio, Lewis Taylor, David R. Greaves, Asif J. Iqbal

**Affiliations:** ^1^Sir William Dunn School of Pathology, University of Oxford, Oxford, United Kingdom; ^2^College of Medical and Dental Sciences, Institute of Cardiovascular Sciences, University of Birmingham, Birmingham, United Kingdom

**Keywords:** CCRL2, chemerin, G protein-coupled receptor, inflammation, chemokines, neutrophils

## Abstract

Chemerin is a chemotactic protein that induces migration of several immune cells including macrophages, immature dendritic cells, and NK cells. Chemerin binds to three G protein-coupled receptors (GPCRs), including CCRL2. The exact function of CCRL2 remains unclear. CCRL2 expression is rapidly upregulated during inflammation, but it lacks the intracellular DRYLAIV motif required for classical GPCR downstream signalling pathways, and it has not been reported to internalise chemerin upon binding. The aim of this study was to investigate what role if any CCRL2 plays during acute inflammation. Using the zymosan- and thioglycollate-induced murine models of acute inflammation, we report that mice deficient in the *Ccrl2* gene display exaggerated local and systemic inflammatory responses, characterised by increased myeloid cell recruitment. This amplified myeloid cell recruitment was associated with increased chemerin and CXCL1 levels. Furthermore, we report that the inflammatory phenotype observed in these mice is dependent upon elevated levels of endogenous chemerin. Antibody neutralisation of chemerin activity in *Ccrl2*^−/−^ mice abrogated the amplified inflammatory responses. Importantly, chemerin did not directly recruit myeloid cells but rather increased the production of other chemotactic proteins such as CXCL1. Administration of recombinant chemerin to wild-type mice before inflammatory challenge recapitulated the increased myeloid cell recruitment and inflammatory mediator production observed in *Ccrl2*^−/−^ mice. We have demonstrated that the absence of CCRL2 results in increased levels of local and systemic chemerin levels and exacerbated inflammatory responses during acute inflammatory challenge. These results further highlight the importance of chemerin as a therapeutic target in inflammatory diseases.

## Introduction

Understanding the endogenous pathways that regulate inflammatory responses is of critical importance for the development of novel therapies for chronic inflammatory disease. Recent approaches for the treatment of inflammatory diseases have focussed around blocking specific inflammatory mediators such as the cytokines TNF-α, IL-1β, and IL-6 (1–3). Whilst such approaches have been very effective for certain diseases [e.g., anti-TNF-α monoclonal antibodies for the treatment of rheumatoid arthritis (RA)] a large percentage of patients either do not respond to treatment or become refractory to therapeutic antibody treatment (4, 5). It is known that cytokines play a central role in shaping the immune response to invading pathogens and in the context of chronic inflammation. It is now appreciated that there exists a plethora of lipid mediators as well as other immune modulating proteins and peptides that play important roles during both the onset as well as the resolution of inflammation (6–8). Annexin-A1 and its N-terminal peptide ac2-26 have been demonstrated to exert potent pro-resolution properties in multiple inflammatory disease models (8, 9). Similarly, a number of lipid mediators including the resolvins, lipoxins, maresins, and protectins have been more recently come to the fore (7, 8). These novel immune modulatory mediators and others may represent new avenues to explore for the treatment of chronic inflammatory disease.

Murine chemerin is a 16 kDa protein produced and secreted as an inactive precursor, pro-chemerin, predominantly by hepatocytes and adipocytes (10). Pro-chemerin is present in the liver, spleen, skin, and plasma (11, 12). During an inflammatory response, inflammatory proteases produced locally by granulocytes and the coagulation cascade, cleave the carboxyl terminus of pro-chemerin to generate active chemerin isoforms at sites of inflammation (13, 14). Chemerin has been implicated in the pathology of a range of inflammatory diseases including RA, inflammatory bowel disease, psoriasis, diabetes, and cardiovascular disease (15–19). However, the exact role played by chemerin and its receptors in inflammatory disease remains unclear.

Our group and others have demonstrated that active chemerin, once generated, is a potent chemoattractant for macrophages, immature dendritic cells (DCs), plasmacytoid dendritic cells (pDCs), and NK cells (20–23). Chemerin binds to three G protein-coupled receptors (GPCRs) with high affinity; CMKLR1, GPR1, and CCRL2 (24). Murine GPR1 is expressed in white adipose tissue, skin, muscle, and in the central nervous system (25). Although chemerin binding to GPR1 has been reported to induce downstream signalling, chemerin is thought to act as a partial agonist at GPR1 whilst chemerin acts as a full agonist at CMKLR1 (26, 27). CMKLR1 is expressed on various immune cells including macrophages, DCs, NK cells, and pDCs (12). It is also expressed on endothelial cells and adipocytes (28, 29). *Cmklr1* mediates the chemotactic effects of chemerin, and its activation has been reported to lead to rapid downstream signalling cascades, which are G_i/0_ coupled (23, 26). The Chemerin/*Cmklr1* axis has been implicated in driving the recruitment of immature DCs, pDCs, and NK cells to local sites of inflammation in a number of inflammatory diseases (22, 30–32). Interestingly, *Cmklr1* has also been reported to play an anti-inflammatory role in a number of inflammatory disease models, although these have predominantly been allergic inflammatory models (12, 33). In addition, our group and others have reported anti-inflammatory effects of synthetic chemerin-derived peptides in a number of inflammation models and these effects seem to be dependent on CMKLR1 (34–36).

CCRL2 is a seven transmembrane receptor that lacks the DRYLAIV intracellular motif required for classical downstream signalling by GPCRs (37). It binds chemerin but does not induce classical downstream signalling nor does it internalise chemerin (20, 37, 38). CCRL2 is expressed on a range of cell types including macrophages, DCs, endothelial cells, and epithelial cells amongst others (38, 39). Expression of CCRL2 is upregulated in response to inflammatory stimuli but the function of CCRL2 during inflammation remains incompletely understood (39, 40). Zabel et al. have proposed a model in which CCRL2 binds to the non-signalling N-terminus of chemerin and then presents it to other cells expressing CMKLR1. In this way, CCRL2 could function to concentrate chemerin at local sites to augment chemerin signalling during inflammation (38). The aim of this study was to further explore the role of the non-signalling chemerin receptor CCRL2 during a self-resolving model of acute inflammation.

We report, for the first time, that animals lacking the chemerin receptor CCRL2 displayed exaggerated neutrophil and inflammatory monocyte recruitment in models of acute inflammation. These effects were due in part to increased levels of chemerin, which augmented production of the neutrophil chemoattractant CXCL1, resulting in increased neutrophil recruitment.

## Materials and Methods

### Animals

B6.129-*Ccrl2^tm1Dgen^*/J mice were obtained from Jackson Laboratories (Bar Harbour, ME, USA). These mice, originally produced by Deltagen (San Mateo, CA, USA), were backcrossed for 12 generations onto the C57BL/6J background. All animal studies were conducted with ethical approval from the Dunn School of Pathology Local Ethical Review Committee and in accordance with the UK Home Office regulations (Guidance on the Operation of Animals, Scientific Procedures Act, 1986).

### Reagents

Recombinant murine chemerin (aa17–156) was reconstituted in PBS supplemented with 0.1% BSA. Neutralising anti-chemerin antibody and goat IgG control were reconstituted in PBS. Both chemerin and antibodies were purchased from R&D Systems (Abingdon, UK). Zymosan was purchased from Sigma-Aldrich (Dorset, UK). Recombinant CCL5 was purchased from Peprotech (London, UK). Bio-gel P100 (45–90 µm) fine polyacrylamide beads were obtained from BIO-RAD Laboratories, Hemel Hempstead, Hertfordshire, UK.

### Murine Bone Marrow-Derived Macrophages (BMDMs)

Bone marrow-derived macrophages were generated as previously described (41). Briefly, fresh bone marrow cells from tibiae and femurs of C57BL/6J mice (8–12 weeks) were isolated and cultured in DMEM media supplemented with 10% heat inactivated fetal bovine serum, 10% L929 cell-conditioned media as a source of macrophage colony-stimulating factor, 100 U/ml penicillin, and 100 µg/ml streptomycin for 7 days. A total of 4 × 10^6^ bone marrow cells were seeded into 10 ml of medium in 100 mm Petri dishes (Sterilin, Abergoed, UK.) and on day 3 an additional 5 ml of medium was added. Cells were harvested by gentle agitation to lift cells off surface.

### Human Umbilical Vein Endothelial Cells (HUVEC)

Human umbilical vein endothelial cells were isolated as previously described (42) and frozen for storage in liquid N_2_. HUVEC were thawed and resuspended in endothelial cell growth medium with supplement mix C (PromoCell, Germany), 100 U/ml penicillin, and 100 µg/ml streptomycin and cultured in pre-coated 0.5% gelatin (Sigma) flask/dishes in a 37°C 5% CO_2_ incubator.

### Cell Activation Assays

Cells were seeded into 6-well plates at a concentration of 1 × 10^6^/ml. They were then exposed to TLR ligands and cytokines for 16 h in a 37°C 5% CO_2_ incubator as described previously. The TLR ligands and cytokines were added to cells at a final concentration of: LPS 100 µg/ml, IFNγ 20 ng/ml, Poly I:C 10 µg/ml, zymosan 10 µg/ml, flagellin 500 ng/ml, IL-4 20 ng/ml, and IL-13 20 ng/ml.

### RNA Isolation and Reverse Transcription and RT-PCR

Total RNA was extracted using the QIAGEN RNeasy Mini kit as instructed by the manufacturer as described previously (43). RNA concentration and purity was determined using ND-1000 spectrophotometer (Nanodrop, Thermo Scientific) at 260/280 and 260/230 nm. cDNA was synthesised from 500 to 800 ng of total RNA using QuantiTect Reverse Transcription kit following the manufacturer’s instructions. cDNA was amplified for 15 min at 42°C and then 3 min at 95°C. Real-time quantitative PCR was performed using Sybr Select Master Mix (Applied Biosystems, Life Technologies) in the Step One Plus Real-time PCR System (Applied Biosystems). All primers were from QuantiTect Primer Assay (Qiagen). The thermal profile included an initiation step for 2 min at 50 and 95°C followed by 40× cycles of 15 s at 95°C and 1 min at 60°C. Cycle threshold values were determined by the StepOne software v2.3 and the mRNA content of samples was inferred by normalising to the housekeeping β-actin gene. Relative expression results were plotted as mRNA expression over actin, normalised to basal samples (44).

### Zymosan or Thioglycollate Induced Peritonitis

Male *Ccrl2*^−^*^/^*^−^ or littermate controls were injected i.p. (0.5 ml) with 100 µg zymosan resuspended in PBS, or 1 ml of 4% thioglycollate (Thioglycollate brewers yeast; Sigma-Aldrich, Dorset, UK) prepared as described previously (45). Mice were sacrificed at specified time points and peritoneal cavities were lavaged with 5 ml ice-cold PBS supplemented with 2 mM EDTA. Blood was collected from the hepatic portal vein into EDTA-coated vacutainers and centrifuged at 2,000 × *g* for 20 min at 4°C to obtain plasma.

### Modulation of Chemerin Levels *In Vivo*

C57BL/6J mice were treated with either recombinant murine chemerin (4 µg) or PBS i.p. for 1 h before zymosan challenge for 4 h. Animals were sacrificed and peritoneal lavage was collected as before. *Ccrl2*^−^*^/^*^−^ mice were pretreated for 24 h with either 100 ng of isotype control IgG antibody or 100 ng of anti-chemerin polyclonal antibody i.p. This was followed by a second injection of isotype control IgG or anti-chemerin antibody 1 h before challenge with zymosan. Mice were sacrificed 4 h later and peritoneal lavage fluid was collected as before.

### Flow Cytometry

Peritoneal exudate cells (PECs) were resuspended in fluorescence-activated cell sorting (FACS) buffer (PBS; 2% FCS, 25 mM HEPES, 5 mM EDTA) containing CD16/CD32 FCγIIR blocking antibody (eBioscience). Specific staining with the following antibodies was performed with appropriate isotype controls; F4/80 (AbD Serotec, clone CI:A3-1), Ly6B.2 (AbDSerotec, Clone 7/4), Ly6 G (BioLegend, clone 1A8), CD11b (BioLegend, clone M1/70), CD115 (BioLegend, clone AFS98), CD4 (BioLegend, clone RM4-5), CD3 (BioLegend, clone 17A2), B220 (eBioscience, clone RA3-6B2), CD8 (BD Pharminogen, clone 53-6.7), and analysed by flow cytometry. Cells were counted using CountBright Absolute Counting Beads (Life Technologies). Cells were analysed with a Dako Cyan ADP flow cytometer (Beckman Coulter Ltd., High Wycombe, UK) and FlowJo software V10 (Tree Star Incorporation, Ashland, OR, USA). Monocytes and neutrophils in the peritoneum were defined as CD45^+^, 7/4^+^, Ly6G^−^ and CD45^+^, 7/4^+^, Ly6G^+^, respectively (46, 47). Blood was collected *via* hepatic portal vein into EDTA-coated vacutainers. Blood was treated in the same manner as the PECs, but red blood cells were lysed after antibody staining using BD FACS Lysing Solution (Buffered solution with <15% formaldehyde and <50% diethylene glycol) before fixation. Ly6C^hi^ blood monocytes were defined as CD45^+^, CD11b^+^, CD115^+^, Ly6C^hi^. Ly6C^lo^ monocytes were defined as CD45^+^, CD11b^+^, CD115^+^, Ly6C^lo^ (48, 49).

### Fluorescence-Activated Cell Sorting

Male C57BL/6J mice were injected i.p. (0.5 ml) with 100 µg zymosan resuspended in PBS. Steady state and zymosan challenged mice were sacrificed 4 h later, and peritoneal cavities were lavaged with 5 ml ice-cold PBS supplemented with 2 mM EDTA. PECs were stained for flow cytometry as described previously. Peritoneal macrophages, monocytes, and neutrophils were FACS sorted using a Beckman Astrios cell sorter directly into RLT buffer for RNA isolation using the QIAGEN RNeasy Mini kit.

### Detection of Secreted Protein by ELISA and Luminex

CXCL1, CCL2, IL-6, and chemerin in peritoneal exudate fluid and plasma were detected using ELISA (R&D Systems, Abingdon, UK). Sandwich ELISAs for chemerin, IL-6, CCL2, and CXCL1 were performed according to manufacturer’s instructions. Custom multiplex polyacrylamide bead assays were purchased from R&D Systems to determine levels of CCL3, CCL4, IL-10, CXCL10, and MMP9 in peritoneal exudate fluid. Briefly, colour-coded beads were pre-coated with antibodies against the targets of interest. Biotinylated detection antibodies specific for each analyte were added, followed by phycoerythrin (PE)-conjugated streptavidin. Samples were read using a laser detection system, which quantifies the amount of PE present for each analyte. The 96-well plates were read on a Bio-Rad Bioanalyser with Bio-Plex Manager software (Hemel Hempstead, Hertfordshire, UK).

### ACEA xCELLigence Chemotaxis Assay

8- to 10-week-old male *Ccrl2*^−^*^/^*^−^ or littermate controls were injected i.p. with 1 ml 2% Bio-gel (P100 Fine, 45–90 µm). PECs (mixture of inflammatory macrophages and neutrophils) were isolated by peritoneal lavage with ice-cold PBS supplemented with 2 mM EDTA 4 days later. Real-time chemotaxis assays were performed using the ACEA RTCA-DP instrument as described previously (50, 51). Briefly, vehicle, chemerin, or recombinant murine CCL5 (160 µl) at 5 nM final concentration was added to the lower chamber of a CIM-16 plate. The upper chamber was attached, and 50 µl of prewarmed chemotaxis buffer added to each of the upper chambers. Following equilibration for 30 min, the plate was transferred into the RTCA-DP system. Bio-gel elicited PECs (50 µl—4 × 10^5^ cells/well), were then added to all upper wells. Cell index (CI) measurements were then taken every 5 s over the 3–4 h assay period. Chemotactic responses were assessed by quantifying the slope of the curve in the first 40 min and by the maximum CI minus minimum CI (Max–Min) values of the curve over the entire experiment.

### CMKLR1 β-Arrestin Recruitment Assays

Recruitment of β-Arrestin to Cmklr1 was measured using the Discoverx PathHunter^®^ eXpress β-Arrestin GPCR Assay following the manufacturer’s protocol. Briefly, CHO-K1 cells stably expressing murine Cmklr1 were seeded into 1/2 area 96-well plates (15,000 cells/well) and incubated at 37°C, 5% CO_2_ for 48 h. Cells were then treated with either vehicle (Cell assay reagent) or indicated concentrations of anti-chemerin antibody for 45 min at 37°C, 5% CO_2_ before stimulation with vehicle or recombinant chemerin (20 nM) for 90 min at 37°C, 5% CO_2_. Cells were lysed and detection of total recruited β-arrestin was determined following the manufacturers protocol and as previously described (51). Luminescence measurements were taken using a PHERAstar microplate reader (BMG Labtech).

### Statistical Analysis

All quantitative data are reported as mean ± SEM of *n* independent biological replicates. Statistical significance was assessed using a Student’s unpaired *t*-test, one-way or two-way analysis of variance (ANOVA) with Dunnett’s multiple comparison *post hoc* test (Prism 6 GraphPad Software, San Diego, CA, USA), *P* < 0.05 was taken to be statistically significant.

## Results

### Chemerin Levels and *Ccrl2* Expression Are Increased during Acute Inflammation

**Figure 1:**
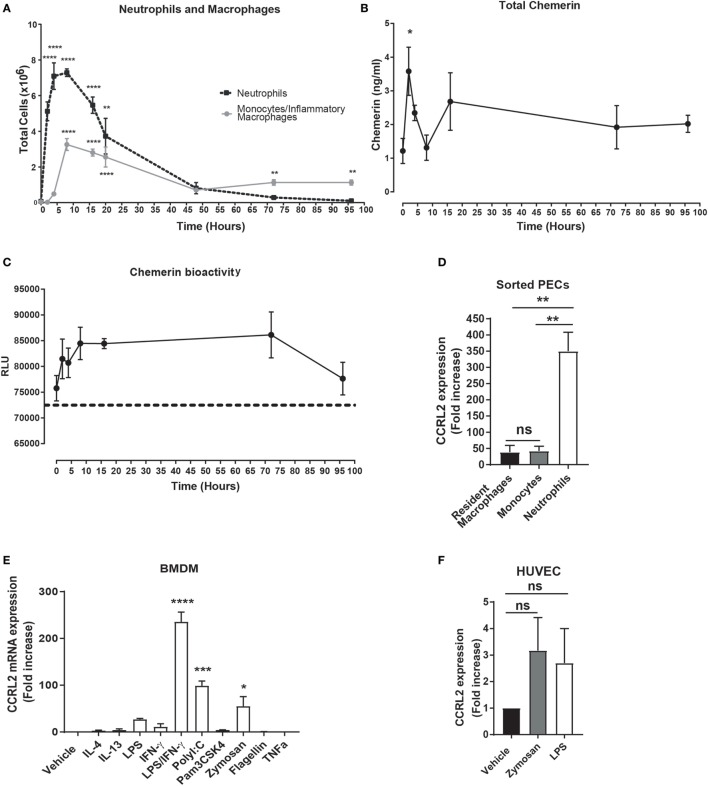
Chemerin bioactivity and *Ccrl2* expression are increased during inflammation. C57BL/6J male mice were injected i.p. with 100 µg zymosan. Animals were sacrificed at indicated time points, and peritoneal cavities were lavaged with ice-cold PBS supplemented with 2 mM EDTA. **(A)** Total neutrophils (black broken line) and inflammatory monocytes/macrophages (grey line) at indicated time points. **(B)** Total chemerin levels from the peritoneal exudate fluid were measured by ELISA at the indicated time points. **(C)** Chemerin bioactivity at the *Cmklr1* receptor was measured in the peritoneal exudate fluid using CHO-K1 cells stably transfected with murine Cmklr1. Cmklr1 activity was assessed by quantification of β-arrestin recruitment to Cmklr1 as measured by luminescence. Dashed line represents background luminance. RLU, relative light units. Error bars represent SEM. *n* = 4–15 mice per time point and *n* = 4 independent experiments. Statistical significance was assessed using one-way analysis of variance (ANOVA) with Dunnett’s multiple comparison *post hoc* test. **(D)** C57BL/6J male mice were injected i.p. with 100 µg zymosan, and 4 h later steady state or zymosan challenged mice were sacrificed, and peritoneal cavities were lavaged with ice-cold PBS supplemented with 2 mM EDTA. Resident peritoneal macrophages from steady state mice and recruited neutrophils and monocytes were sorted by fluorescence-activated cell sorting into RLT buffer, and *Ccrl2* mRNA expression was assessed by qPCR. Error bars represent SEM of *n* = 3 mice/group. **(E,F)** mRNA expression of *Ccrl2* receptor was analysed by qPCR on bone marrow-derived macrophages (BMDMs) and human umbilical vein endothelial cells (HUVECs) following exposure to TLR ligands and cytokines for 16 h. Error bars represent SEM of *n* = 2 separate experiments. Statistical significance was assessed using one-way ANOVA with Dunnett’s multiple comparison *post hoc* test. *P ≤ 0.05, **P ≤ 0.01, ***P ≤ 0.001, ****P ≤ 0.0001.

We and others have previously demonstrated that i.p. challenge with zymosan induces robust inflammatory mediator production in the peritoneum as well as inflammatory cell recruitment (47). Injection of 100 µg zymosan resulted in robust inflammatory cell recruitment with neutrophils peaking at 4 h post zymosan challenge and monocytes peaking at 8 h (Figure 1A). The role played by chemerin during inflammation remains incompletely understood. To interrogate this in our model of acute inflammation, we quantified total chemerin levels in the peritoneum during this acute inflammatory response and found that levels were significantly increased compared with naïve mice 4 h following zymosan challenge (2.5 ± 0.4 ng/ml at 4 h compared with 1.2 ± 0.3 ng/ml in naïve mice) (Figure 1B). One caveat using an ELISA to measure chemerin levels is that it is a quantification of total chemerin levels, including inactive pro-chemerin and potentially shorter bioactive chemerin isoforms generated during inflammation. To investigate chemerin bioactivity during this acute inflammatory response, we compared the ability of the peritoneal exudate fluid from animals injected with zymosan at different time points to activate the CMKLR1 chemerin receptor on CMKLR1 transfected CHO-K1 cells (Figure 1C). Using this bioassay, chemerin bioactivity progressively increased after zymosan injection peaking at 8 h and decreasing to baseline levels by 96 h (Figure 1C). These data suggest that although total chemerin levels as measured by ELISA peaked at 4 h and decreased rapidly by 8 h, chemerin bioactivity continued to increase up to 8 h and remained increased up to 72 h post zymosan injection.

The function of CCRL2 during inflammation remains to be fully elucidated, but the expression of CCRL2 has been reported to be increased during inflammation (39). To confirm and extend this observation, we FACS sorted resident peritoneal macrophages as well as recruited peritoneal neutrophils and monocytes from wild-type (WT) C57BL/6J mice following a 4 h zymosan challenge. We found that *Ccrl2* was expressed on three cell types but expression was highest on recruited neutrophils following zymosan challenge (Figure 1D). In addition, we cultured BMDMs and HUVECs and challenged them with a range of inflammatory stimuli (Figures 1E,F). Addition of zymosan, LPS, interferon-γ, poly I:C all increased BMDM expression of *Ccrl2* with the combination of LPS and interferon-γ resulting in the largest increase in expression (~235-fold increase compared with vehicle) (Figure 1E). Zymosan and LPS also increased *Ccrl2* expression in HUVECs (Figure 1F). These results are in agreement with the published literature, indicating that *Ccrl2* expression is indeed rapidly upregulated on a number of cells during acute inflammation (39, 40).

### *Ccrl2*^−*/*−^ Mice Displayed Increased Monocyte and Neutrophil Recruitment to the Inflamed Peritoneum

**Figure 2:**
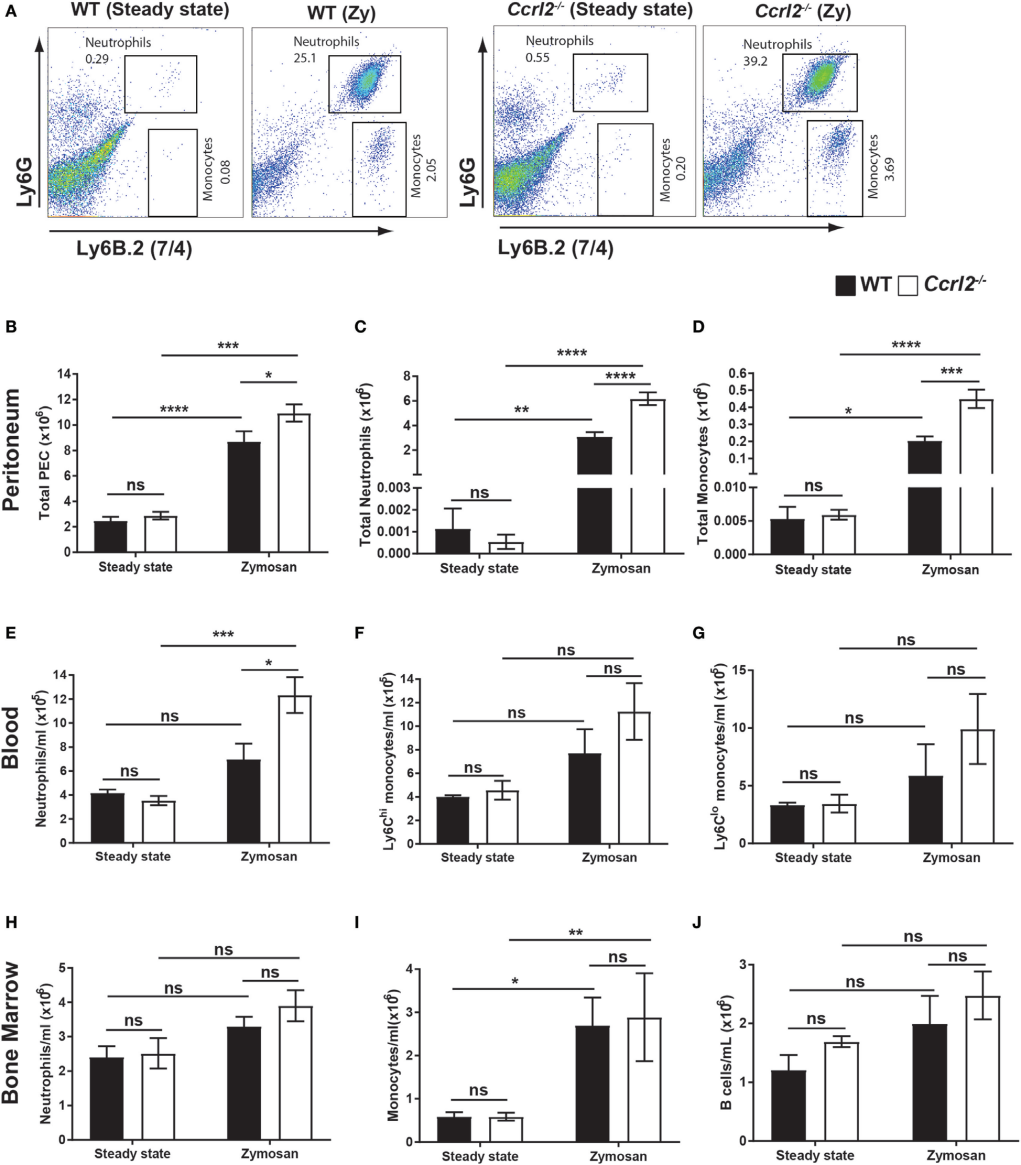
Deletion of *Ccrl2* increased neutrophil mobilisation and recruitment to local sites of inflammation. 8- to 10-week-old male *Ccrl2*^−^*^/^*^−^ or age-matched littermate controls were injected with zymosan i.p. (100 µg/animal), and 4 h later, zymosan injected animals or steady state animals were sacrificed. Peritoneal cavities were lavaged with 5 ml ice-cold PBS supplemented with 2 mM EDTA. Cells were quantified using counting beads, and cell populations were analysed using flow cytometry. **(A)** Representative flow cytometry plots of the peritoneal cavities of wild-type (WT) and *Ccrl2*^−^*^/^*^−^ steady state mice or mice challenged with zymosan. Monocytes were defined as Ly6B.2 (7/4)^hi^, Ly6G^lo^ and neutrophils were defined as Ly6B.2 (7/4)^hi^, Ly6G^hi^. WT animals are presented on the left and *Ccrl2*^−/−^ animals are presented on the right. **(B–D)** Total peritoneal cell counts from steady state animals or from animals 4 h post zymosan challenge. Error bars represent SEM of *n* = 4–13 animals/group and *n* = 2 independent experiments. **(E–G)** Blood cell counts in WT and *Ccrl2*^−^*^/^*^−^ mice. Error bars represent SEM. *n* = 4–9 animals/group and *n* = 2 independent experiments. **(H–J)** Quantified bone marrow cells. Error bars represent SEM. *n* = 4–9 animals/group from *n* = 2 independent experiments. Statistical significance was assessed using a two-way analysis of variance with Dunnett’s *post hoc* multiple comparisons test *P ≤ 0.05, **P ≤ 0.01, ***P ≤ 0.001, ****P ≤ 0.0001.

To investigate the potential functional consequence of upregulation of Ccrl2 during acute inflammation *in vivo*, we challenged WT mice or *Ccrl2*^−/−^ mice with 100 µg zymosan for 4 h and quantified inflammatory cell recruitment and mobilisation. Zymosan challenge resulted in significantly more total cells recruited to the peritoneum of *Ccrl2*^−^*^/^*^−^ mice compared with WT after 4 h (Figure 2B). There was also a striking increase in monocyte recruitment (0.4 ± 0.05 × 10^6^ in *Ccrl2*^−/−^ mice compared with 0.2 ± 0.03 × 10^6^ in WT mice) and neutrophil recruitment (6.2 ± 0.52 × 10^6^ in *Ccrl2*^−/−^ mice compared with 3.1 ± 0.35 × 10^6^ in WT mice) to the peritoneum of *Ccrl2*^−^*^/^*^−^ mice (Figures 2A–D). Other cell populations including CD4 T cells, CD8 T cells and B cells were also quantified but no significant differences were observed between the two genotypes (data not shown).

We next examined the blood from these animals 4 h post zymosan challenge to investigate whether the increased monocyte and neutrophil recruitment we observed at the site of inflammation was mirrored in the blood, indicating increased mobilisation of innate immune cells from bone marrow and spleen. Similarly to the peritoneum, there was a twofold increase in blood neutrophils in *Ccrl2*^−^*^/^*^−^ mice challenged with zymosan compared with WT (Figure 2E). There was also an increase in Ly6C^hi^ monocytes, although this was not significant (Figure 2F). In agreement with the results from the peritoneum, there were no differences in circulating B cells, CD4 T cells or CD8 T cells between these genotypes (data not shown).

Since we observed increased neutrophil and monocyte numbers in the peritoneum and blood of *Ccrl2*^−^*^/^*^−^ mice following zymosan challenge, we investigated if the increased numbers of systemic leucocytes was associated with differences in levels in the bone marrow (Figures 2H–J). *Ccrl2*^−^*^/^*^−^ did not display any significant differences in total bone marrow neutrophils (Figure 2H) monocytes (Figure 2I) or B cells (Figure 2J). Collectively, our results suggest that the elevated neutrophil levels observed in the peritoneum are the result of increased initial systemic inflammatory responses of *Ccrl2*^−^*^/^*^−^ mice.

Intriguingly, Ccrl2 appears to be primarily important in the initial stages of the acute inflammatory response. When we investigated later time points following zymosan challenge (48 h), we found no significant differences in neutrophil or monocyte numbers in the peritoneum or blood between *Ccrl2*^−/−^ mice and WT mice (Figures S2B,C,E,F in Supplementary Material). In addition, there were no significant differences in local or systemic chemerin levels at this late time point (Figures S2D,G in Supplementary Material).

### Steady State *Ccrl2*^−*/*−^ Mice Displayed No Obvious Alterations in Resident Leucocyte Populations in Any Tissues Examined

To establish whether the increased inflammatory cell recruitment observed in *Ccrl2*^−^*^/^*^−^ mice during zymosan challenge could be explained by increased circulating myeloid cells under resting conditions, we undertook a phenotypic analysis of the peritoneum, blood, bone marrow and spleen of unchallenged WT and *Ccrl2*^−^*^/^*^−^ mice (Figure 2). We found no differences in neutrophils or monocytes between the two groups (Figures 2C,D). There were no differences in Ly6C^hi^ monocytes, Ly6C^lo^ monocytes or neutrophils in the blood between the two groups (Figures 2E–G). Similarly, there were no differences in monocytes, neutrophils or B cells in the bone marrow (Figures 2H–J). Nor were there obvious differences in the spleens under homeostatic conditions (data not shown). Finally, we endeavoured to assay basal levels of CXCL1 and IL-6 in the peritoneal lavage fluid of these mice, but all samples tested were below the limit of detection (set at 15 pg/ml) of the assays (data not shown). These results indicate that the increased neutrophil and monocyte recruitment to the peritoneum seen following zymosan challenge were due to differences in initial inflammatory responses rather than a constitutive increase in circulating neutrophil and monocyte numbers.

### *Ccrl2*^−*/*−^ Mice Displayed Increased CXCL1 and Chemerin Levels at Early Time Points following Zymosan Challenge

**Figure 3:**
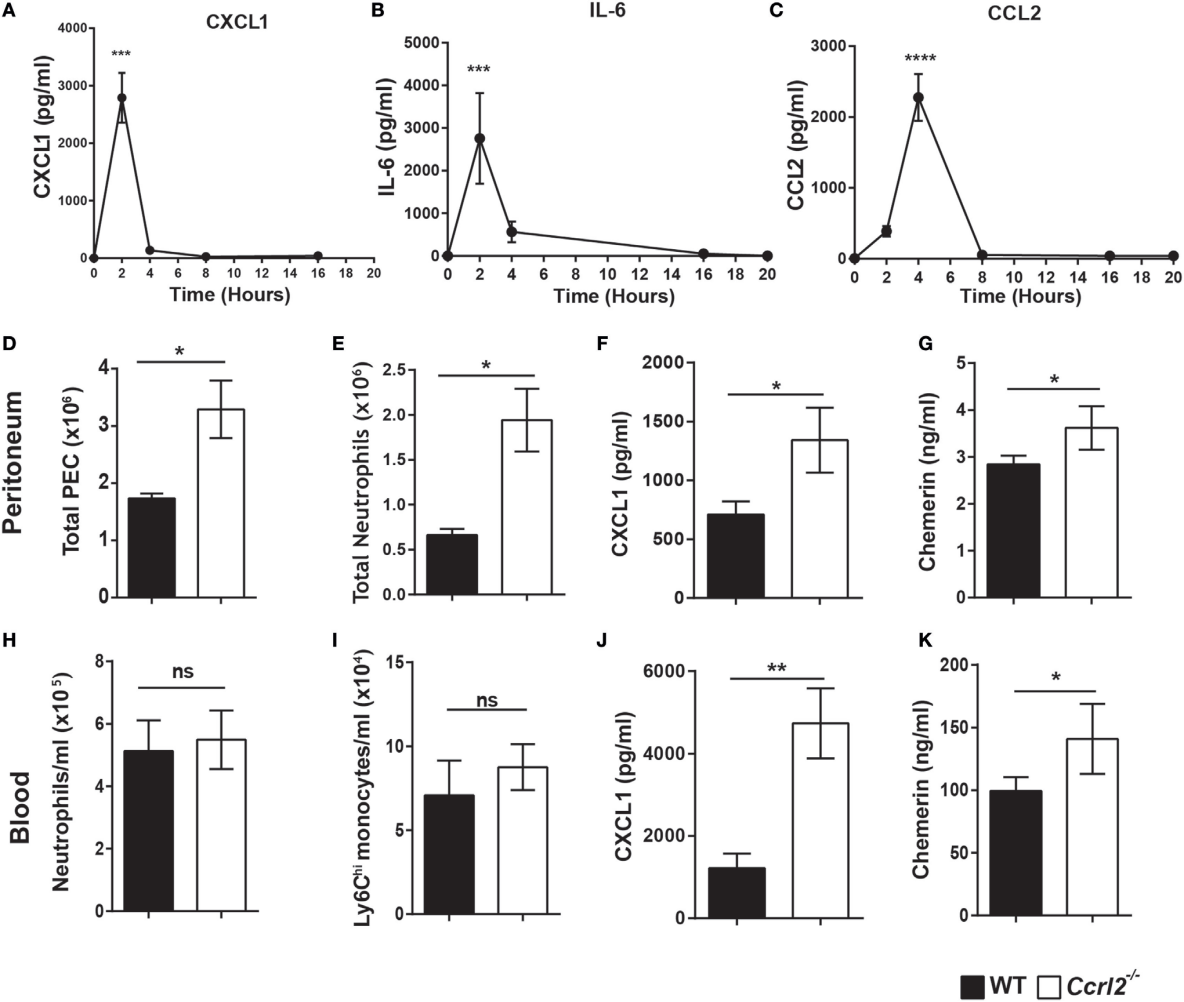
Increased neutrophil recruitment in *Ccrl2*^−^*^/^*^−^ mice was associated with increased CXCL1 and chemerin levels at early time points. **(A–C)** 8- to 10-week-old male C57BL/6J wild-type (WT) mice were injected with zymosan i.p. (100 µg/animal), and animals were sacrificed at indicated time points. Peritoneal cavities were lavaged with ice-cold PBS supplemented with 2 mM EDTA. Mediator levels were quantified by ELISA. Error bars represent SEM. *n* = 4–15 mice per time point and *n* = 2 independent experiments. Statistical significance was assessed using one-way analysis of variance with Dunnett’s multiple comparisons *post hoc* test. **(D–L)** 8- to 10-week-old male *Ccrl2*^−^*^/^*^−^ or age-matched littermate control mice were injected with zymosan i.p. (100 µg/animal), and animals were sacrificed 2 h later. Cells were quantified using counting beads, and cell populations were analysed using flow cytometry. **(D,E)** Total peritoneal cell counts following 2 h zymosan challenge. **(F)** CXCL1 and chemerin **(G)** levels in the peritoneum of *Ccrl2*^−^*^/^*^−^ and WT mice quantified by ELISA. **(H,I)** Blood neutrophils and monocytes in WT and *Ccrl2*^−^*^/^*^−^ mice. **(J,K)** Plasma levels of CXCL1 and chemerin quantified by ELISA. Data are presented as mean ± SEM. *n* = 8 animals/group and *n* = 2 independent experiments. Statistical significance was assessed using a Student’s unpaired *t*-test. *P ≤ 0.05, **P ≤ 0.01.

Having shown that mice lacking the CCRL2 chemerin receptor displayed exaggerated acute inflammatory responses following zymosan challenge, we next investigated if the increased monocyte and neutrophil recruitment was due to elevated chemokine or inflammatory mediator levels. There were no significant differences in mediator levels between the two genotypes at the 4 h time point (Table 1). However, a number of mediators including CXCL1 (an important neutrophil chemoattractant) and IL-6 displayed rapid induction following zymosan challenge peaking at 2 h (Figures 3A,B).

**Table 1:** Local mediators (pg/ml) produced in peritoneal exudate cell fluid following 4 h challenge with 100 µg zymosan i.p. as measured by Luminex.

	IL-6	CCL2	CCL3	CCL4	CXCL1	CXCL2	CXCL10

	ns	ns	ns	ns	ns	ns	ns
Wild type	759 ± 123	1,218 ± 81	34.50 ± 5.3	744.3 ± 41	78.6 ± 6.4	506 ± 166	620 ± 50
*Ccrl2*^−/−^	705 ± 83	1,013 ± 21	26.40 ± 2.1	736.8 ± 32	127.8 ± 28	400 ± 148	525 ± 24

*Data are presented as mean ± SEM of n = 8 animals*.

*Statistical significance was assessed by a Student’s unpaired t-test, ns = P > 0.05*.

Since CXCL1 and IL-6 levels peaked rapidly following zymosan insult, we challenged both *Ccrl2*^−/−^ mice and littermate controls with zymosan for 2 h and assessed the resulting inflammatory responses. Similar to the 4 h time point, we observed significantly more neutrophils recruited to the peritoneum in the *Ccrl2*^−^*^/^*^−^ mice compared with WT (Figure 3E). There were very few if any monocytes recruited to the peritoneum at this early time point (Figure 1B). We observed a twofold increase in CXCL1 levels in the peritoneum of these animals as well as significantly elevated chemerin levels (Figures 3F,G). Whilst we observed no obvious differences in monocyte or neutrophil numbers in the blood (Figures 2H,I), there was a threefold increase in CXCL1 plasma levels (Figure 3K). Chemerin levels were also significantly elevated in the blood of the *Ccrl2*^−/−^ mice compared with WT at the 2 h time point (Figure 3L).

### *Ccrl2*^−/−^ Mice Displayed Increased Neutrophil Numbers in the Peritoneum after Thioglycollate Challenge

**Figure 4:**
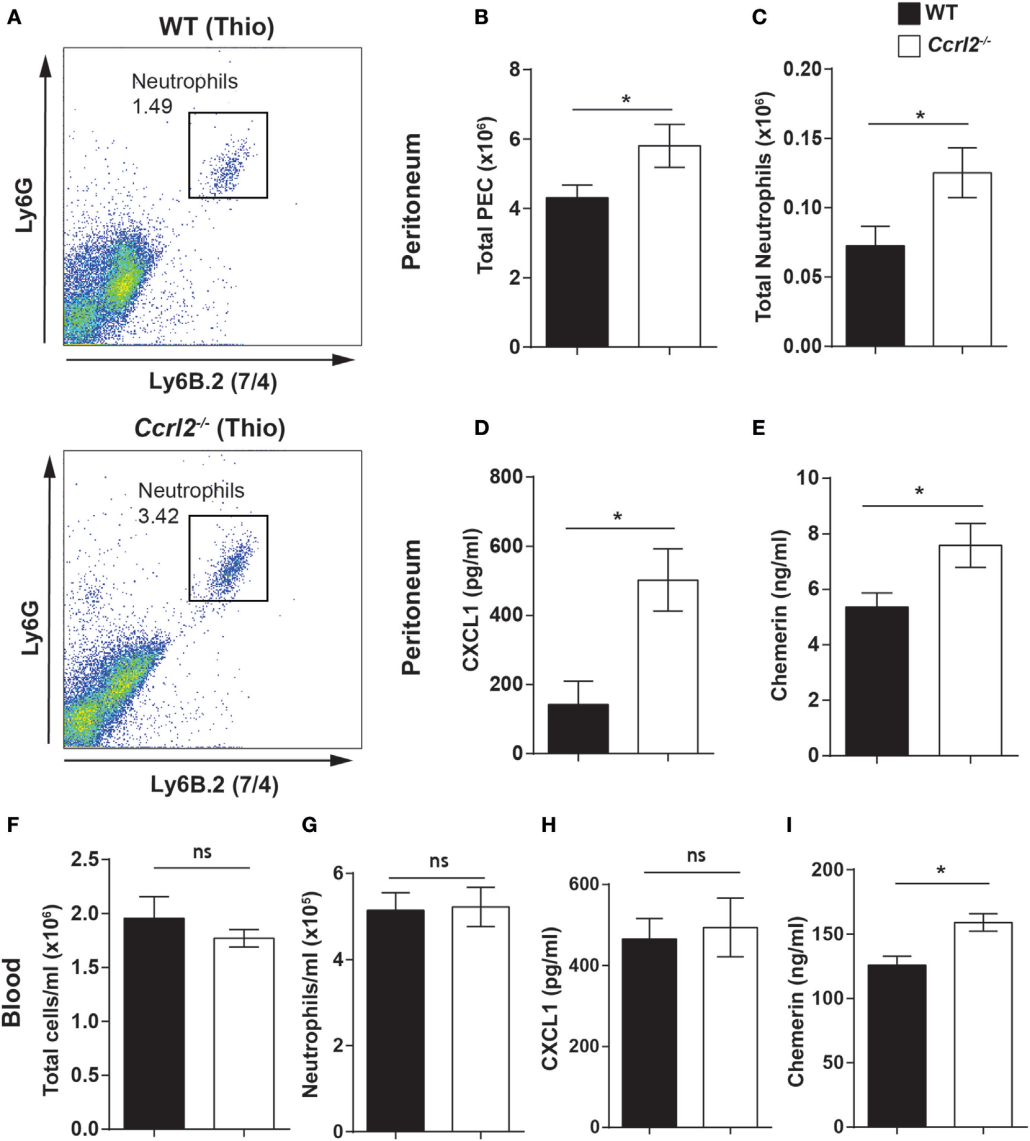
Mice lacking *Ccrl2* displayed exaggerated neutrophil recruitment during acute inflammation independently of stimulus. 8- to 10-week-old male *Ccrl2*^−^*^/^*^−^ mice or age-matched littermate controls were injected with 4% thioglycollate, and 1 h later, animals were sacrificed. Peritoneal cavities were lavaged with ice-cold PBS supplemented with 2 mM EDTA. Cells were quantified using counting beads, and cell populations were analysed using flow cytometry. **(A)** Representative flow cytometry plots of the peritoneal cavities of wild-type (WT) and *Ccrl2*^−^*^/^*^−^ mice treated with thioglycollate. Neutrophils were defined as Ly6B.2 (7/4)^hi^ and Ly6G^hi^ **(B,C)** Total peritoneal cell counts following 1-h thioglycollate challenge. **(D)** CXCL1 and chemerin **(E)** levels in the peritoneum of *Ccrl2*^−^*^/^*^−^ and WT mice quantified by ELISA. Total blood leucocytes **(F)** and neutrophils **(G)** in WT and *Ccrl2*^−^*^/^*^−^ mice. **(H)** Plasma CXCL1 and chemerin **(I)** levels in *Ccrl2*^−^*^/^*^−^ and WT quantified by ELISA. Mean ± SEM. *n* = 5 animals/group and *n* = 1 experiment. Statistical significance was assessed using a Student’s unpaired *t*-test. *P ≤ 0.05.

To explore if the increased neutrophil recruitment observed in the *Ccrl2*^−^*^/^*^−^ mice was also evident with other inflammatory stimuli, we challenged these mice i.p. with thioglycollate. Thioglycollate is a well-established inflammatory stimulus used to elicit inflammatory macrophages (after 4–5 days) (45, 52). However, it can also be used to interrogate neutrophil recruitment in the initial stages of inflammation. Following a 1 h challenge with 4% thioglycollate, we observed a small but distinct population of neutrophils recruited to the peritoneum (Figure 4A). We chose this time point because it represents an early stage of neutrophil recruitment in response to thioglycollate. *Ccrl2*^−^*^/^*^−^ mice displayed a twofold increase in neutrophil recruitment to the peritoneum compared with WT controls (0.07 ± 0.01 × 10^6^ neutrophils in WT compared with 0.13 ± 0.01 × 10^6^ neutrophils in *Ccrl2*^−/−^ mice) (Figure 4C). There were also significantly higher CXCL1 and chemerin levels in the peritoneum of the *Ccrl2*^−^*^/^*^−^ mice compared with WT mice at this early time point (Figures 4D,E).

When we examined the blood of these mice, there was no difference in neutrophil numbers between *Ccrl2*^−^*^/^*^−^ mice and WT controls (Figures 4F,G). This is perhaps not surprising given the early time point tested. There were, however, elevated chemerin levels in the blood of the *Ccrl2*^−^*^/^*^−^ mice compared with WT controls (Figure 4I). As we did not observe any differences in neutrophil numbers in the blood of these animals, it seems unlikely there would be changes in the bone marrow at this early time point but this was not assessed (53). Collectively, these observations demonstrate that mice lacking *Ccrl2* display exaggerated neutrophil recruitment to local sites of inflammation as well as elevated chemerin and CXCL1 levels irrespective of the stimulus used to elicit the response.

### Neutralisation of Endogenous Chemerin in *Ccrl2*^−*/*−^ Mice Abrogated the Exaggerated Inflammatory Phenotype

**Figure 5:**
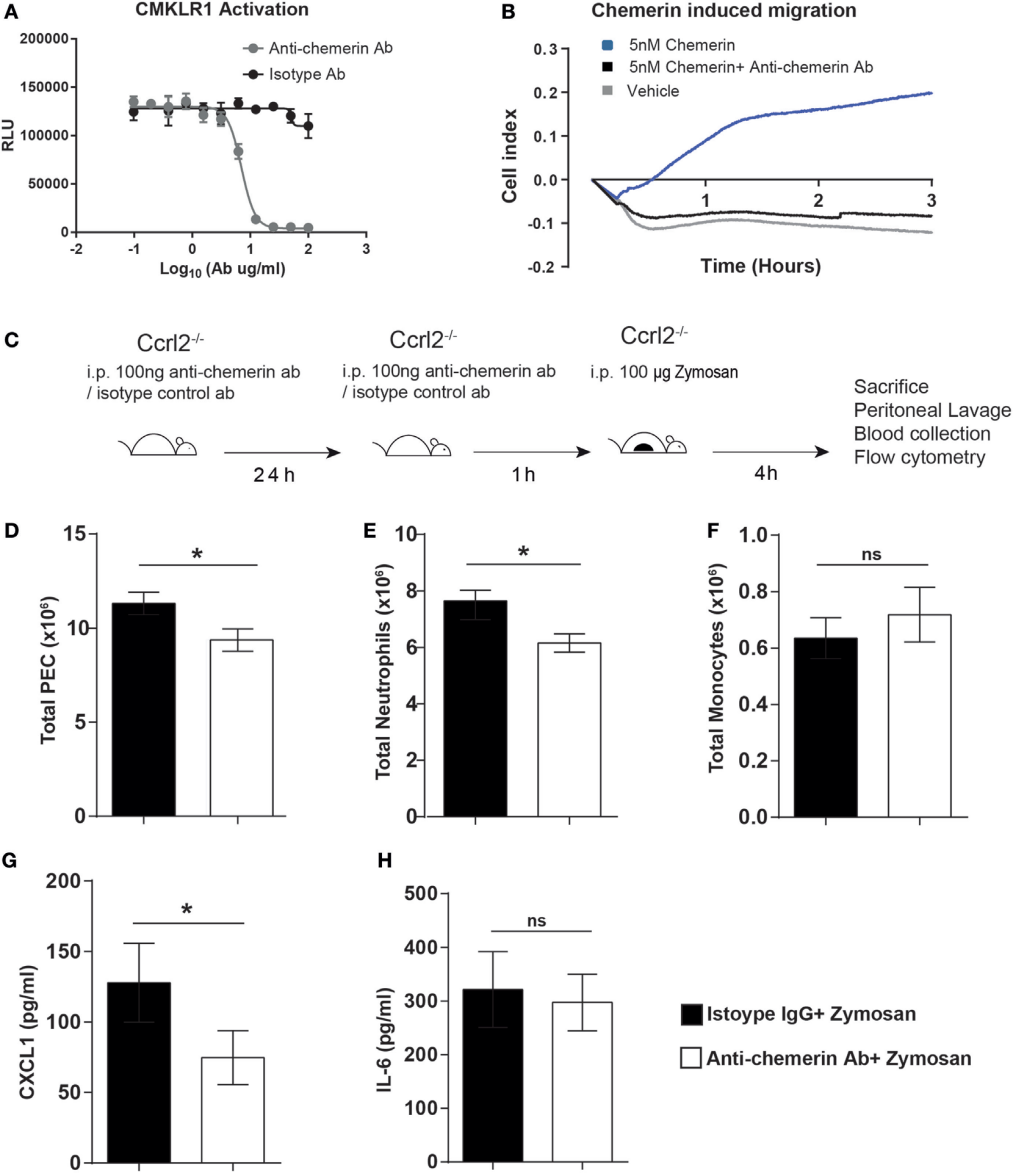
Treatment with an anti-chemerin blocking antibody attenuated the exaggerated inflammatory responses observed in *Ccrl2*^−^*^/^*^−^ mice. **(A)** CHO-K1 cells stably transfected with murine Cmklr1 were plated out in a 96-well plate for 48 h before stimulation. Cells were challenged with indicated concentrations of anti-chemerin antibody or isotype control before challenge with 20 nM murine chemerin. *Cmklr1* activity was assessed by quantification of β-arrestin recruitment to Cmklr1 as measured by luminescence. RLU, relative light units. Error bars = SD of three technical replicates of one experiment. **(B)** Representative real-time chemotaxis trace of biogel-elicited peritoneal exudate cells (PECs). 8- to 10-week-old male C57BL/6J mice were injected i.p. with 2% Bio-gel (polyacrylamide beads) and sacrificed 4 days later. Peritoneal cavities were lavaged with ice-cold PBS supplemented with 2 mM EDTA. Cells were pretreated with vehicle or anti-chemerin antibody (ab) for 45 min. A gradient of 5 nM of the 5 nM chemerin was allowed to form, and chemotaxis was measured of 4 × 10^5^ cells (400,000 cells/well) for 3 h. **(C)** Schematic of *in vivo* experimental design. **(D–G)** 8- to 10-week-old male *Ccrl2*^−^*^/^*^−^ mice were pretreated with 100 ng of anti-chemerin blocking antibody or isotype control IgG antibody i.p. for 24 h before challenge with zymosan for 4 h. Animals were sacrificed, and peritoneal cavities were lavaged with ice-cold PBS supplemented with 2 mM EDTA. Cells were quantified using counting beads, and cell populations were analysed using flow cytometry **(D)** Total cells. **(E)** Total neutrophils. **(F)**. Total monocytes. Levels of CXCL1 **(G)** and IL-6 **(H)** in the peritoneum of indicated groups were quantified by ELISA. Mean ± SEM. *n* = 2–8 mice/group. Statistical significance was assessed using a Student’s unpaired *t*-test. *P ≤ 0.05, **P ≤ 0.01.

From our previous experiments, *Ccrl2*^−^*^/^*^−^ mice displayed increased myeloid cell recruitment in short-term models of acute inflammation. This was associated with elevated CXCL1 and chemerin levels. Given our group and others have identified the CCRL2 ligand chemerin, as an important modulator of inflammation and chemotaxis, it seemed plausible that the elevated levels of chemerin during acute inflammation in *Ccrl2*^−^*^/^*^−^ mice was responsible for the increased myeloid cell recruitment (20, 21, 23). To test this hypothesis, we used a blocking anti-chemerin antibody to neutralise endogenous chemerin levels in the *Ccrl2*^−^*^/^*^−^ mice before zymosan challenge (Figure 5). We first confirmed that this antibody could indeed block signalling at the Cmklr1 receptor. Using Cmklr1 transfected CHO-K1 cells, we demonstrated that preincubation with the antibody efficiently blocked chemerin induced β-arrestin recruitment to Cmklr1 (Figure 5A). We also tested the antibody in primary cells and confirmed that it effectively blocked chemerin-induced chemotaxis of biogel-elicited macrophages in a real-time chemotaxis system (Figure 5B). Following a 24 h pretreatment with isotype control or anti-chemerin antibody, *Ccrl2*^−/−^ mice were challenged for 4 h with zymosan as before (Figure 5C). Animals that received the anti-chemerin antibody displayed significantly less total leucocyte and neutrophil recruitment to the peritoneum compared with mice that received the isotype control (Figures 5D,E; Figure S1 in Supplementary Material). There was no significant effect on monocyte recruitment (Figure 5F). When we measured mediator levels in the peritoneum of these mice, we observed a twofold reduction in CXCL1 levels in the anti-chemerin treated mice compared with isotype control treated mice and no differences in IL-6 levels between the groups (Figures 5G,H). These results indicate that the elevated chemerin levels observed in *Ccrl2*^−^*^/^*^−^ mice contribute to the exaggerated leucocyte recruitment as well as the elevated CXCL1 levels observed in these mice during acute inflammation.

### Chemerin, the Ligand for CCRL2, Increased Neutrophil Recruitment in WT Mice

**Figure 6:**
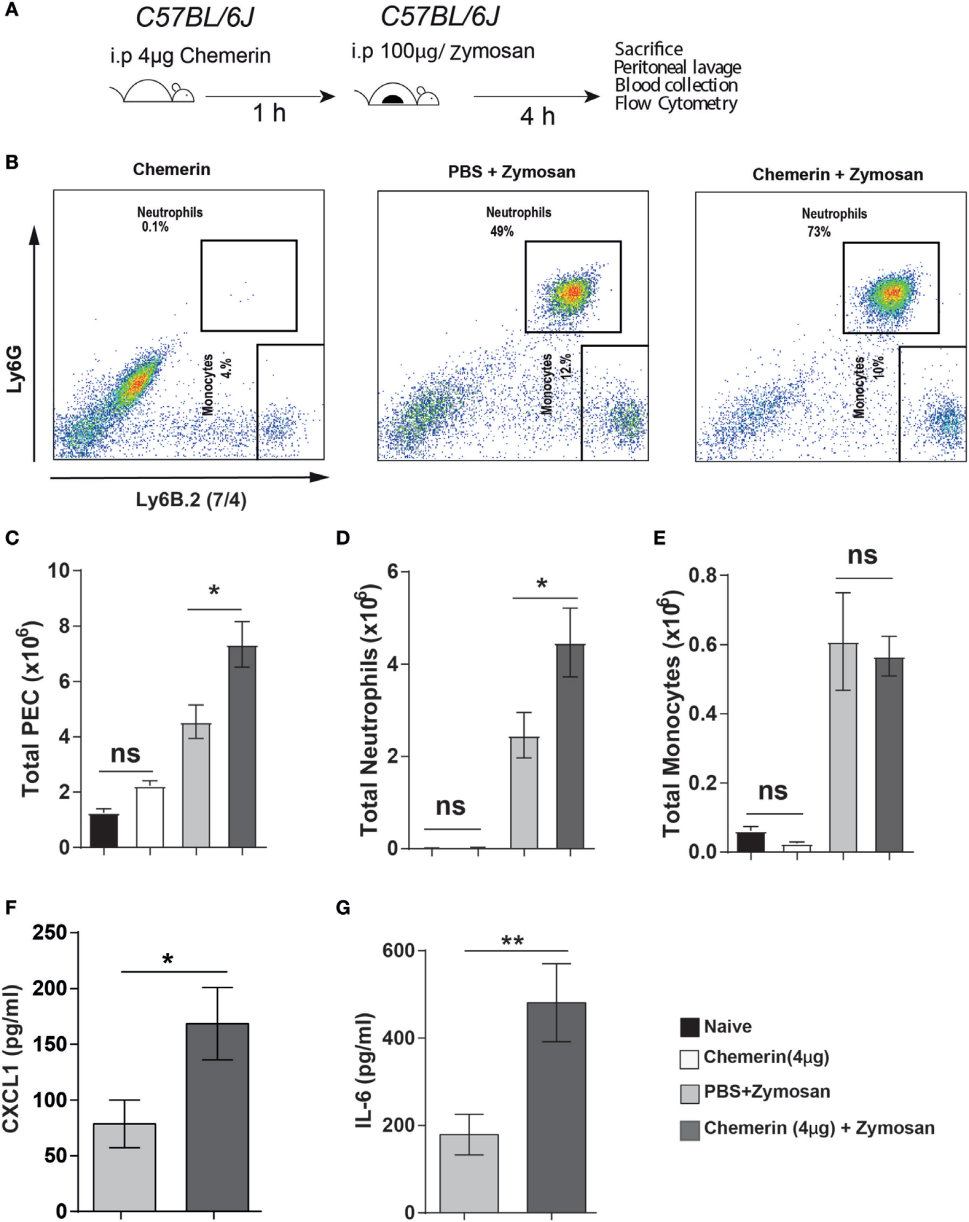
Recombinant chemerin pretreatment increased inflammatory cell recruitment in mice during acute inflammation. **(A)** 8- to 10-week-old male C57BL/6J mice were pretreated (i.p.) with recombinant murine chemerin (4 µg/mouse) or PBS for 1 h before challenge with zymosan i.p. (100 μg/mouse). 4 h later, mice were sacrificed, and peritoneal cavities were lavaged with ice-cold PBS supplemented with 2 mM EDTA. Cells were quantified using counting beads, and cell populations were analysed using flow cytometry. **(B)** Representative flow cytometry plots of the peritoneal cavities of wild-type and *Ccrl2*^−^*^/^*^−^ mice challenged with indicated treatments. Monocytes were defined as Ly6B.2 (7/4)^hi^, Ly6G^lo^ and neutrophils were defined as Ly6B.2 (7/4)^hi^, Ly6G^hi^. **(C)** Total CD45^+^ leucocytes recruited to the peritoneum of indicated groups following zymosan challenge. **(D)** Total neutrophils recruited to the peritoneum of indicated groups following zymosan challenge. **(E)** Total monocytes recruited to the peritoneum of indicated groups following zymosan challenge. Error bars represent SEM. *n* = 3–7 mice/group and *n* = 2 independent experiments. Levels of CXCL1 **(F)** and IL-6 **(G)** in the peritoneum of indicated groups. Mean ± SEM. *n* = 6–7 mice/group. Statistical significance was assessed using a Student’s unpaired *t*-test. *P ≤ 0.05, **P ≤ 0.01.

Chemerin has previously been reported to induce the expression of various inflammatory cytokines and chemokines (including CXCL1 and CCL2) in epithelial and endothelial cells (54, 55). This provides a possible mechanism by which the higher chemerin levels in *Ccrl2*^−/−^ mice could cause increased CXCL1 levels during an inflammatory response. To further investigate the role of chemerin in exacerbating inflammation in our model, we pretreated WT mice with recombinant murine chemerin (4 µg/mouse) for 1 h before zymosan challenge (Figures 6A,B). Mice that received chemerin pretreatment displayed increased total cell recruitment to the peritoneum compared with mice that received PBS (4.5 × 10^6^ leucocytes in PBS treated animals compared with 7.5 × 10^6^ leucocytes in chemerin pretreated) (Figure 6C). Chemerin pretreated mice also displayed increased neutrophil recruitment to the peritoneum compared with PBS pretreated mice (2.5 ± 0.5 × 10^6^ neutrophils in zymosan alone compared with 4.5 ± 0.7 × 10^6^ in chemerin pretreated) (Figure 6D). We did not observe any differences in monocyte recruitment between the groups (Figure 6E). Importantly, injection of chemerin alone did not result in any neutrophil or monocyte recruitment (Figures 6B–E). When we analysed the inflammatory exudate from these mice, we found that chemerin pretreated mice had significantly higher levels of CXCL1 and IL-6 (Figures 6F,G). Mice that were treated with chemerin displayed a 2-fold and 2.6-fold increase in CXCL1 and IL-6 levels, respectively. We also observed similar effects using a lower dose of 0.5 µg chemerin per mouse as a pretreatment before zymosan challenge (data not shown).

### Increased Recruitment of Neutrophils and Monocytes in *Ccrl2*^−*/*−^ Mice Was Not due to a Direct Effect of CCRL2 on Chemerin Induced Chemotaxis

**Figure 7:**
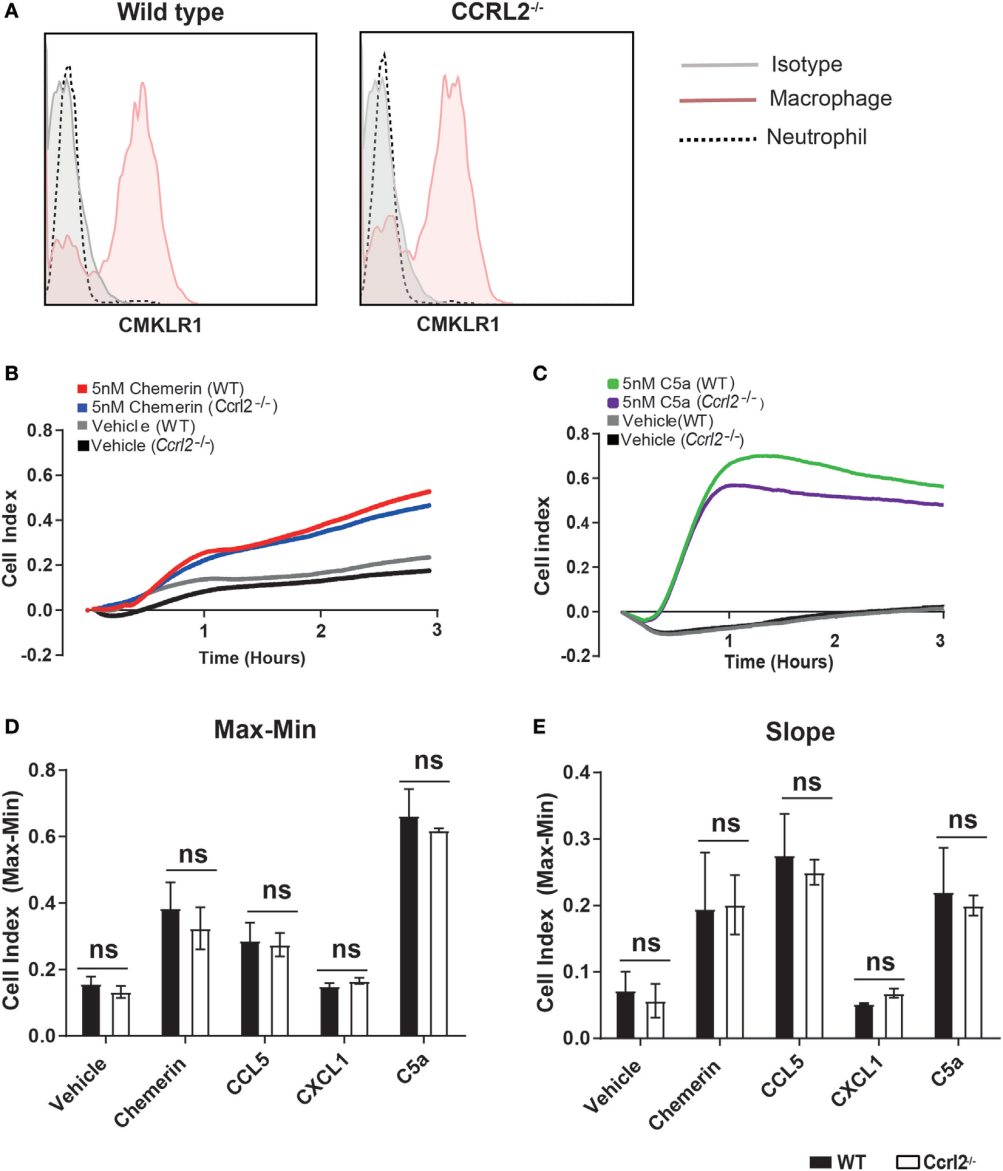
Absence of *Ccrl2* had no effect on the migratory behaviour of leucocytes towards macrophage or neutrophil chemoattractants. 8- to 10-week-old male *Ccrl2*^−^*^/^*^−^ mice and age-matched littermate controls were injected i.p. with 2% Bio-gel (polyacrylamide beads) and sacrificed 4 days later. Peritoneal cavities were lavaged with ice-cold PBS supplemented with 2 mM EDTA. **(A)** Representative histogram cytometry plots displaying Cmklr1 expression on biogel elicited macrophages and neutrophils. Macrophages were defined as F4/80^hi^, Ly6B.2 (7/4)^lo^, Ly6G^lo^, neutrophils were defined as Ly6B.2 (7/4)^hi^, Ly6G^hi^. **(B,E)** A gradient of 5 nM of the indicated chemoattractant was allowed to form, and chemotaxis was measured of 4 × 10^5^ cells (400,000 cells/well) for 3 h. Representative chemotaxis traces of wild type and *Ccrl2*^−^*^/^*^−^ peritoneal exudate cell chemotaxis to 5 nM chemerin **(B)** or 5 nM C5a. **(C)**. **(D)** Max–Min analysis and **(E)** slope analysis. Error bars are SEM of four experiments with independent macrophage preparations. Significance was assessed using two-way analysis of variance with Sidak’s multiple comparison test. ns = *P* > 0.05.

One possible explanation for the observed differences in inflammatory cell recruitment was that lack of Ccrl2 may in someway alter the migratory behaviour of inflammatory cells in response to chemerin or other chemotactic ligands. To test this, we used Bio-gel elicited PECs, which we recently demonstrated to be a mixture of inflammatory macrophages and neutrophils (50, 51). We first demonstrated that Bio-gel elicited neutrophils do not express the chemerin Cmklr1 receptor (Figure 7A). Using the real-time chemotaxis platform, we demonstrated that deletion of CCRL2 had no appreciable effect on PEC migration towards chemerin, CCL5, CXCL1, or C5a, ruling out any direct effect of CCRL2 on cell migration (Figures 7B–E).

## Discussion

In this study, we have demonstrated for the first time that Ccrl2 plays a non-redundant role in dampening the recruitment of myeloid cells to local sites of acute inflammation. We report that Ccrl2 is expressed on resident peritoneal macrophages as well as recruited monocytes and neutrophils following zymosan challenge (Figure 1D). Interestingly, the highest levels of expression appear to be on recruited neutrophils and this is in agreement with a recent report by Del Prete et al. (56). In addition, *Ccrl2* expression on macrophages and endothelial cells is increased after exposure to inflammatory stimuli. This is in agreement with published reports (Figures 1E,F) (39, 40). After 4 h of zymosan challenge we observed that mice lacking expression of the Ccrl2 chemerin receptor displayed a twofold increase in monocyte and neutrophil recruitment to the peritoneum (Figures 2B,C). These effects of Ccrl2 were not localised only to the site of inflammation, as we also observed increased neutrophil numbers in the blood, suggesting more systemic effect (Figure 2E). These systemic effects were predominantly associated with neutrophil recruitment as we did not observe significant differences in monocyte numbers in the blood or bone marrow (Figures 2F,I). The fact that there were significant differences only in local monocyte numbers but not in systemic numbers may be a feature of the early time point evaluated here, as we know from our kinetic studies that monocyte numbers peak later (Figure 1B). Importantly, at an earlier time point (2 h) in which we could more easily evaluate cytokine and chemokine levels, we observed that mice lacking Ccrl2 displayed higher levels of CXCL1 in the peritoneum (Figure 3F). The elevated levels of CXCL1 both locally and systemically would explain why the majority of differences we observe in the *Ccrl2*^−/−^ mice were in the context of neutrophil migration. This phenotype was consistently associated with higher endogenous levels of chemerin in *Ccrl2*^−/−^ mice and was abrogated after blocking chemerin activity with an anti-chemerin antibody (Figure 5). Furthermore, this phenotype was recapitulated in WT mice by injection of recombinant murine chemerin, further indicating a role for chemerin in driving this exaggerated neutrophil recruitment (Figure 6). Finally, this exaggerated inflammatory phenotype of *Ccrl2*^−/−^ mice appears to be predominantly associated with the initial stages of acute inflammatory responses. Absence of Ccrl2 did not appear to affect neutrophil or monocyte numbers locally or systemically at later time points (Figure S2 in Supplementary Material).

Ccrl2 is one of the less studied chemerin receptors and arguably performs the least obvious function. It binds chemerin but there is no reported downstream signalling upon receptor ligation in primary cells (38, 39). Whilst it was initially thought to serve a similar function to the decoy receptors ACKR1 (or DARC) and ACKR2 (or D6), which dampen inflammation by binding and internalising inflammatory chemokines, Ccrl2 does not internalise chemerin or any other chemokines (38, 57). Whilst the exact role of Ccrl2 during inflammation has yet to be fully elucidated, its expression has been documented by a number of groups including in this report (Figures 1E,F) to be rapidly upregulated during inflammation, suggesting a conserved function required during inflammatory responses (39, 40).

Several studies have endeavoured to clarify the role played by Ccrl2 *in vivo* but there have been conflicting results in different experimental models. Zabel et al. demonstrated that mice lacking Ccrl2 displayed reduced ear swelling and leucocyte influx in a mast cell dependent model of atopic allergy. The exact mechanism by which Ccrl2 modulated these responses was not clear. However, cells expressing Ccrl2 were shown to bind and concentrate chemerin locally *in vitro*. When these cells were incubated with *Cmklr1* transfected cells, they induced calcium flux, indicating a possible role for Ccrl2 in chemerin presentation (38). In the *in vitro* model, Ccrl2 expressing cells were capable of binding chemerin and presenting it to Cmklr1 expressing cells when in close proximity. It was postulated this mode of action played a role in mast cell responses to low dose IgE challenge. However, this seems unlikely to be the case in our model of acute inflammation. Administration of recombinant bioactive chemerin to WT mice exacerbated the acute inflammatory response (Figure 6). If Ccrl2 were functioning to increase chemerin signalling, which appears to be pro-inflammatory here, one would expect to observe reduced inflammation following zymosan challenge in *Ccrl2*^−/−^ mice. Yet in Figures 2 and 3, we observed the converse. An alternative hypothesis, therefore, is that *in vivo*, increased expression of Ccrl2 on other cell types during inflammation such as vascular endothelial cells may enable binding of free chemerin, which in turn reduces systemic levels of chemerin (39). This would thereby decrease the chemerin available to interact with Cmklr1 expressing cells and therefore dampen the ensuing pro-inflammatory responses elicited by chemerin as described by Neves et al. (55).

Mazzon et al. reported that mice lacking Ccrl2 displayed exacerbated disease in a model of experimental autoimmune encephalitis (EAE) as well as increased *Ccrl2* expression on mononuclear cells in WT mice during EAE (58). The authors reported that in mice lacking Ccrl2, T cells, and macrophages were further polarised towards an inflammatory phenotype during disease, but the mechanism by which deletion of the *Ccrl2* gene enhanced disease remained unexplained (58).

At the time of writing, the same group more recently reported a new study in which mice lacking Ccrl2 were protected in two murine models of arthritis in contrast to their previous study using the EAE model, highlighting the complexity of the chemerin/Ccrl2 axis (56, 58). Importantly, the authors report that the protective effect of deletion of *Ccrl2* in these models was due to defects in neutrophil recruitment and trafficking due to CXCR2 heterodimersastion with Ccrl2. These results seem to be at odds with our current findings but possibly reflect differences in the disease models used to interrogate this biology. The kinetics and disease pathology differ significantly between the acute models of inflammation used in our study and the more chronic disease models used by Del Prete et al. in their most recent report (56). Clearly, further studies will be necessary to clarify the exact role played by Ccrl2 during inflammation but it seems likely to be disease and even tissue specific (59).

In agreement with an earlier study by Monnier et al. who reported higher chemerin levels in the blood of *Ccrl2*^−/−^ mice following intranasal administration of LPS, we observed elevated chemerin levels in the blood of *Ccrl2*^−/−^ animals following zymosan and thioglycollate challenge (Figures 3 and 4) (39). In addition, using BMDMs from WT animals and HUVECs, we observed increased expression of *Ccrl2* mRNA after stimulation with inflammatory stimuli (up to ~230-fold increase on BMDMs) (Figure 1E). We also report increased neutrophil recruitment and CXCL1 levels both locally and systemically (Figures 2 and 3) in *Ccrl2*^−^*^/^*^−^ mice challenged with 100 µg zymosan. To the best of our knowledge, this is the first report of such an observation. The increased neutrophil recruitment during acute inflammation in these mice can be at least partly explained by increased CXCL1 and chemerin levels. Previous studies from our lab and others have not detected the chemotactic chemerin receptor Cmklr1 on murine neutrophils (23, 56, 60). There has since been one report of murine neutrophils expressing Cmklr1; however, we failed to detect any Cmklr1 expression on murine neutrophils (Figure 7A) (35). It seems unlikely, therefore, that the increased neutrophil recruitment we observed in our model is due to any direct chemotactic effects of chemerin on neutrophils. We did not observe any differences in migration between WT and *Ccrl2*^−/−^ cells in response any mediators tested in agreement with Del Prete et al. (Figures 7C,D) (56). Importantly, the phenotype observed in *Ccrl2*^−^*^/^*^−^ mice is not related to the receptors that detect zymosan (dectin-1, TLR2/6) as we observed a similar phenotype with thioglycollate challenge, which is a more severe inflammatory insult (61). Our results support the hypothesis that Ccrl2 is important for regulating both local and systemic chemerin levels during an acute inflammatory response.

This exacerbated inflammatory response was not simply a feature of increased basal myeloid cell numbers, as we observed no differences in leucocyte populations between the two groups in any tissue tested under steady state conditions (Figure 2). Rather, the increased myeloid cell recruitment in *Ccrl2*^−^*^/^*^−^ mice was associated with increased chemerin and CXCL1 levels both locally and systemically. Chemerin has previously been reported to induce pro-inflammatory signalling in microvascular endothelial and smooth muscle cells (55). Chemerin treatment increased mRNA expression of a number of pro-inflammatory mediators including CCL2, TNF-α, and VCAM-1 (55). Another study by Lin et al. demonstrated that chemerin administration to WT mice induced more severe inflammation in a model of DSS-induced colitis, which was characterised by increased inflammatory cytokines and an inhibition of M2 polarisation of resident macrophages (62). Taken together, these reports support our hypothesis that increased chemerin levels observed in the *Ccrl2*^−^*^/^*^−^ mice were responsible for increased myeloid cell recruitment *via* the increased production of inflammatory mediators such as CXCL1 during sterile peritonitis.

We confirmed the importance of chemerin in the phenotype of *Ccrl2*^−^*^/^*^−^ mice using an anti-chemerin polyclonal blocking antibody to inhibit chemerin signalling in these animals before zymosan challenge (Figure 5). Neutralisation of endogenous chemerin in the *Ccrl2*^−^*^/^*^−^ mice decreased the total leucocytes and neutrophils recruited to the peritoneum compared with isotype controls, indicating that elevated chemerin levels did indeed play a role in the exaggerated neutrophil recruitment observed in these animals (Figures 5C,D). We previously observed that blockade of endogenous chemerin in WT mice resulted in increased neutrophil and monocyte recruitment to the peritoneum of WT mice following challenge with 10 µg zymosan (34). In our current study, we did not interrogate chemerin blockade in WT mice but when chemerin activity was blocked in *Ccrl2*^−/−^ mice during 100 µg zymosan challenge, we observed decreased neutrophil recruitment compared with isotype control treated *Ccrl2*^−/−^ mice. These data highlight further differences in chemerin behaviour depending on the intensity of inflammatory insult and genetic backgrounds used. From the results in our current study, it is plausible that chemerin may be more important for driving neutrophil rather than monocyte recruitment in this acute model of inflammation, as chemerin blockade had no significant effect on monocyte numbers. However, it is known that neutrophils are important for the recruitment of monocytes during an inflammatory response and are capable of secreting a number of monocyte chemoattractants (63, 64). Chemerin bioactivity may not have been completely blocked in these animals, hence although we observed a significant decrease in neutrophil recruitment, this may not been sufficiently blunted to in turn appreciably decrease monocyte recruitment at this time point. When we interrogated mediator levels, we observed a significant decrease in local levels of CXCL1 but no change in IL-6 following blockade of endogenous chemerin (Figures 5F,G). CXCL1 and IL-6 were evaluated as CXCL1 is a key driver of neutrophil recruitment and IL-6 is a systemic marker of inflammation (65–68).

As predicted from our results using chemerin blocking antibodies, chemerin pretreatment of WT mice before zymosan challenge increased total cell and neutrophil recruitment to the peritoneum as well as elevated levels of inflammatory chemokines and cytokines, similar to what we observed in the *Ccrl2*^−^*^/^*^−^ mice (Figure 6). Importantly, injection of chemerin alone did not induce any neutrophil recruitment after 4 h at the dose used (Figures 6B–E). We demonstrate here that full-length bioactive chemerin can increase neutrophil recruitment when administered before an inflammatory stimulus but previous studies from our laboratory have reported that a synthetic chemerin-derived peptide called C15 has anti-inflammatory effects in a similar model (albeit using 10-fold lower dose of zymosan as an inflammatory stimulus) (34). The most likely explanation for this apparent discrepancy is differences in the pharmacology and downstream signalling between the full-length chemerin protein and the 15 amino acid chemerin peptide. The signalling cascades that are activated at the murine Cmklr1 receptor upon chemerin binding for example, are relatively well characterised (involving the MAP kinase, RhoA, ROCK, MEK1/2, and P38 proteins amongst others) (23, 26). However, very little if anything is known about the signalling pathways induced by the C15 peptide. Whilst chemerin is known to act as a potent chemoattactant for macrophages, DCs, and NK cells, C15 does not exhibit similar effects on any cell types investigated thus far (20, 22, 23, 30, 50). Clearly, the full-length protein and the smaller peptide elicit quite different intracellular signalling pathways downstream of the Cmklr1 chemerin receptor and this would suggest they play quite different roles *in vivo*.

We have endeavoured to confirm the requirement for CXCL1 in the exaggerated neutrophil recruitment in *Ccrl2*^−/−^ mice by pretreating WT and *Ccrl2*^−/−^ mice with a blocking anti-CXCL1 antibody before zymosan challenge. However, we did not observe significant decreases in neutrophil recruitment in either group despite previous reports (data not shown) (65). However, these results are perhaps not surprising given the high affinity of these chemokines for their receptors as well as the functional redundancy in the system (69). Hence, we have demonstrated that chemerin is elevated in *Ccrl2*^−/−^ mice and that elevated chemerin is capable of driving the exaggerated neutrophil recruitment and increased levels of CXCL1 during acute inflammation. However, we cannot exclude the possibility that other mediators or pathways are also involved. Further studies will be required to fully clarify this.

In summary, we have demonstrated that the non-signalling chemerin receptor Ccrl2 serves a non-redundant role in dampening acute inflammatory responses *in vivo*. The absence of Ccrl2 resulted in exaggerated acute inflammatory responses as well as elevated CXCL1 and chemerin levels. Blockade of endogenous chemerin in *Ccrl2*^−^*^/^*^−^ mice abrogated the elevated neutrophil recruitment. We observed that administration of recombinant murine chemerin to WT mice induced similar exaggerated inflammatory responses to those seen in *Ccrl2*^−^*^/^*^−^ mice. Importantly, deletion of the *Ccrl2* gene did not have any direct effect on myeloid cell chemotaxis towards chemerin or other chemokines. Our data are consistent with a model in which Ccrl2 serves to bind and maintain chemerin levels below a pathological threshold during acute inflammation and the more severe inflammatory responses observed in *Ccrl2*^−^*^/^*^−^ mice are due to significantly elevated free chemerin levels. The increased chemerin signalling in turn induces higher production of inflammatory chemokines such as CXCL1, which results in elevated myeloid cell recruitment and more severe inflammation. Our experiments suggest that chemerin could be a therapeutic target in the treatment of inflammatory diseases, particularly RA, in which chemerin has consistently been implicated in the pathology of this disease (16, 19, 54, 70).

## Ethics Statement

All animal studies were conducted with ethical approval from the Dunn School of Pathology Local Ethical Review Committee and in accordance with the UK Home Office regulations (Guidance on the Operation of Animals, Scientific Procedures Act, 1986).

## Author Contributions

DR-K, SV, CR, LT, TK, DG, and AI performed experiments; DR-K, SV, and AI analysed results and made the figures; DR-K, SV, AI, and DG designed the research and wrote the paper. All the authors provided critical revision of the manuscript.

## Conflict of Interest Statement

The authors declare that the research was conducted in the absence of any commercial or financial relationships that could be construed as a potential conflict of interest.
