# Supplementary figures and images for: Absence of the Non-Signalling Chemerin Receptor CCRL2 Exacerbates Acute Inflammatory Responses In Vivo
Source: Front Immunol. 2017 Nov 21;8:1621. doi: 10.3389/fimmu.2017.01621 (PMC5702352; doi:10.3389/fimmu.2017.01621)

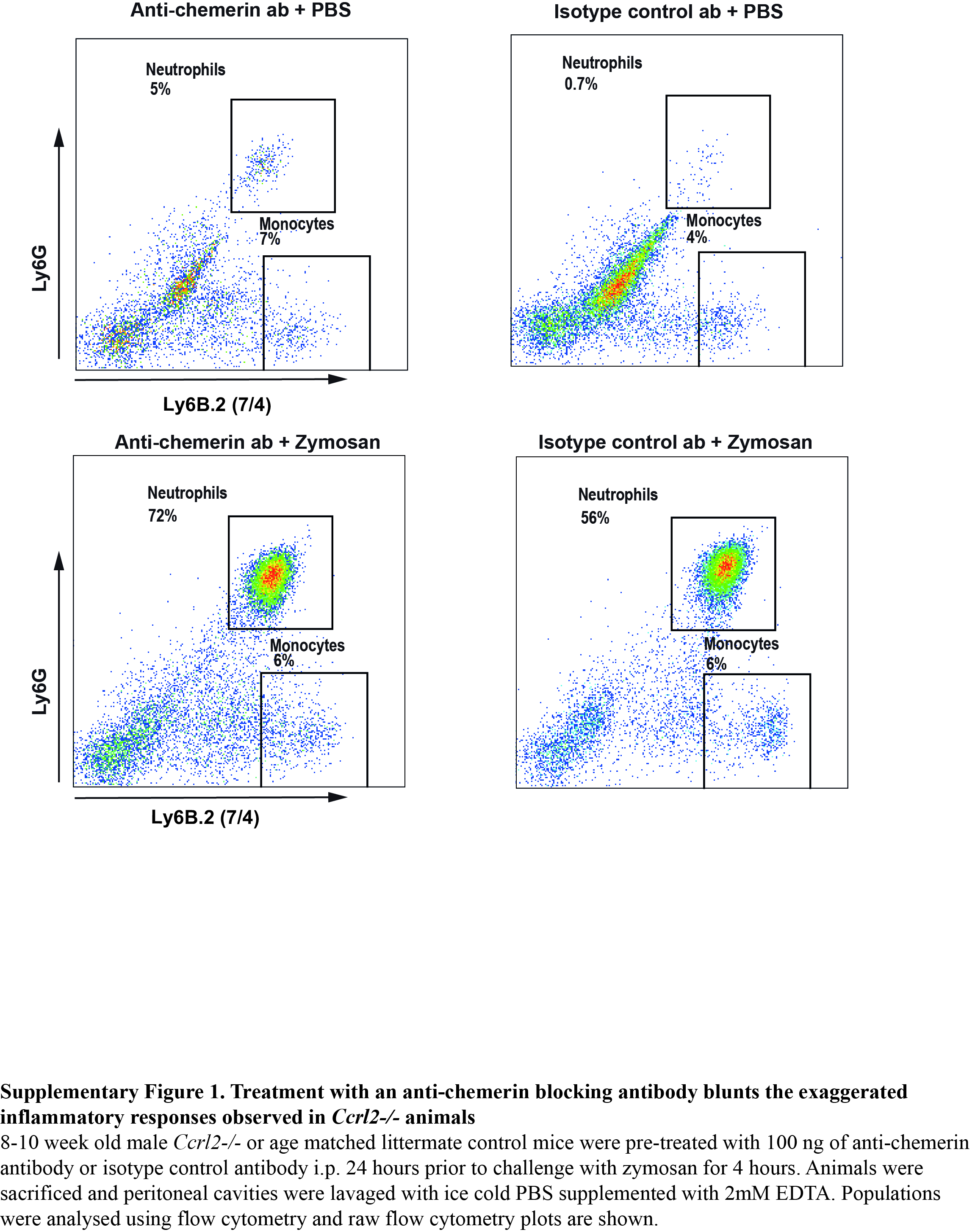

Supplement: Supplementary file 1 [file Image_1.TIF]

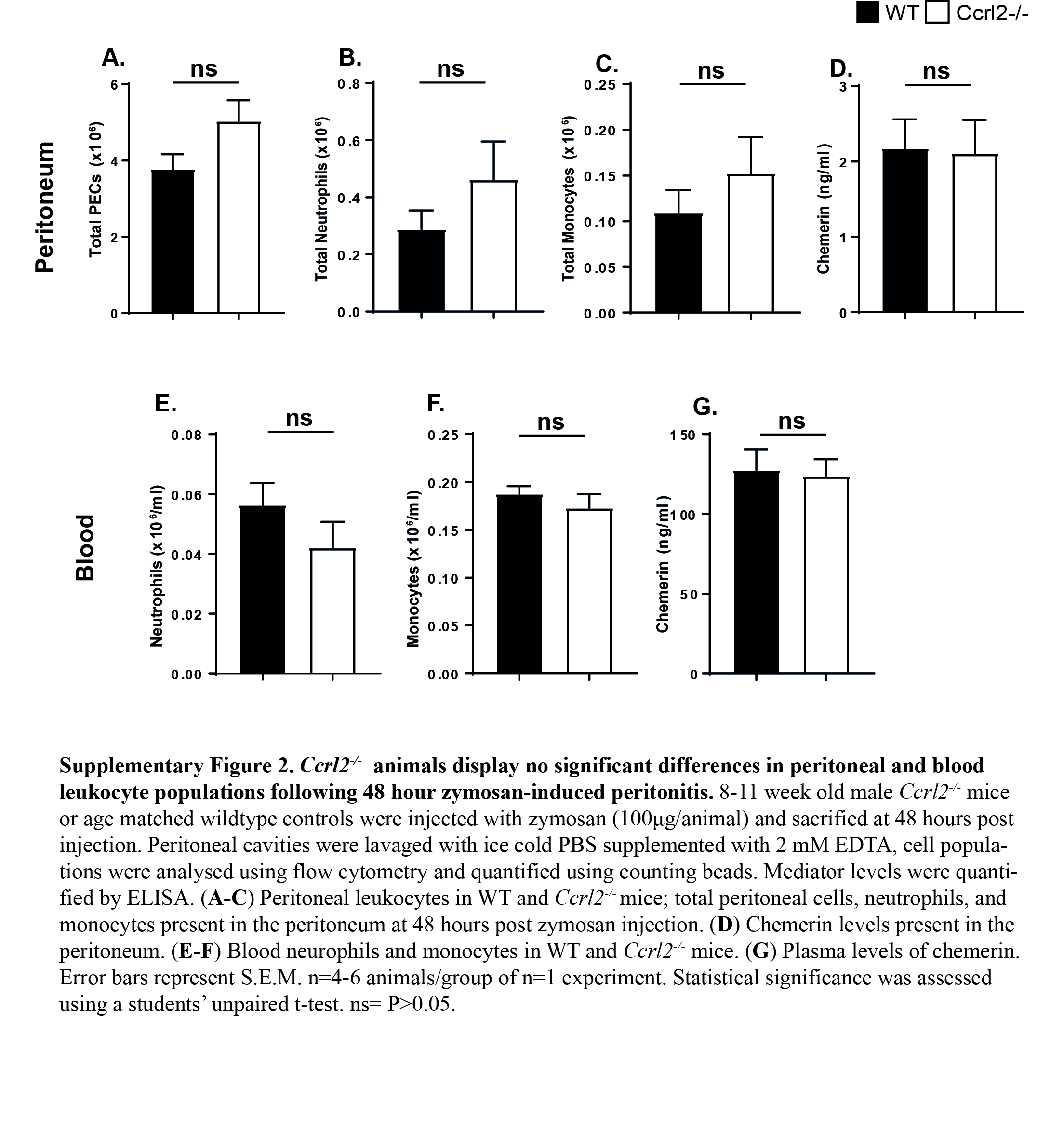

Supplement: Supplementary file 2 [file Image_2.TIF]
